# Examining Neural Connectivity in Schizophrenia Using Task-Based EEG: A Graph Theory Approach

**DOI:** 10.3390/s23218722

**Published:** 2023-10-25

**Authors:** Sergio Iglesias-Parro, María F. Soriano, Antonio J. Ibáñez-Molina, Ana V. Pérez-Matres, Juan Ruiz de Miras

**Affiliations:** 1 Department of Psychology, University of Jaén, 23071 Jaén, Spain; 2 Mental Health Unit, San Agustín Hospital de Linares, 23700 Linares, Spain; 3 Department of Software Engineering, University of Granada, 18071 Granada, Spain

**Keywords:** EEG, graph measures, schizophrenia, mind wandering, on task

## Abstract

Schizophrenia (SZ) is a complex disorder characterized by a range of symptoms and behaviors that have significant consequences for individuals, families, and society in general. Electroencephalography (EEG) is a valuable tool for understanding the neural dynamics and functional abnormalities associated with schizophrenia. Research studies utilizing EEG have identified specific patterns of brain activity in individuals diagnosed with schizophrenia that may reflect disturbances in neural synchronization and information processing in cortical circuits. Considering the temporal dynamics of functional connectivity provides a more comprehensive understanding of brain networks’ organization and how they change during different cognitive states. This temporal perspective would enhance our understanding of the underlying mechanisms of schizophrenia. In the present study, we will use measures based on graph theory to obtain dynamic and static indicators in order to evaluate differences in the functional connectivity of individuals diagnosed with SZ and healthy controls using an ecologically valid task. At the static level, patients showed alterations in their ability to segregate information, particularly in the default mode network (DMN). As for dynamic measures, patients showed reduced values in most metrics (segregation, integration, centrality, and resilience), reflecting a reduced number of dynamic states of brain networks. Our results show the utility of combining static and dynamic indicators of functional connectivity from EEG sensors.

## 1. Introduction

Schizophrenia (SZ) is a heterogeneous disorder characterized by a range of symptoms that can vary in severity and presentation among affected individuals, including positive (e.g., hallucinations and delusions), negative (e.g., blunted affect, alogia, and avolition), and cognitive symptoms (e.g., disorganized speech, thought, and/or attention). These symptoms have substantial consequences for individuals’ lives, affecting various domains such as cognition, employment, education, relationships, and quality of life [1,2]. SZ not only imposes a substantial burden on families and caregivers but also leads to high levels of stress, emotional strain, and disruptions within family dynamics due to its chronic nature and the unpredictability of symptoms [3]. The financial costs associated with treatment, medications, and supportive services further contribute to the burden experienced by families [4]. SZ also has significant social consequences, impacting healthcare systems, public resources, and social welfare programs. The costs of hospitalization, outpatient care, and long-term treatment for individuals diagnosed with SZ pose a substantial economic load [5]. Advancing our understanding of the underlying causes of schizophrenia can lead to improved diagnostic accuracy, personalized treatments, early intervention strategies, prevention programs, and reduced societal burdens. Moreover, research plays a vital role in challenging misconceptions, reducing stigma, and promoting a more inclusive and supportive environment for individuals living with schizophrenia.

Electroencephalography (EEG), while not a standalone diagnostic tool for schizophrenia, offers valuable insights into the disorder’s neural dynamics and functional abnormalities due to its high temporal resolution [6,7,8].

Research studies employing EEG have identified various patterns of brain activity in individuals diagnosed with SZ. These findings include alterations in oscillatory activity, such as increased theta and delta activity, and decreased alpha and beta activity in specific brain regions [9,10,11,12]. These abnormalities may reflect disturbances in neural synchronization and information processing in cortical circuits. Additionally, functional connectivity studies have revealed alterations in the connectivity between brain regions in individuals diagnosed with SZ. Disruptions in large-scale networks, including the default mode network and the fronto-parietal network, have been observed, suggesting impaired integration and coordination of neural activity [13,14,15].

While numerous EEG studies on SZ have utilized resting state data to uncover spontaneous brain activity and connectivity [8,16,17,18,19,20], this paper shifts its focus towards a task-based EEG analysis. Although this approach presents challenges in patient work, it enables the exploration of specific altered cognitive processes in SZ, including working memory deficits and event-related potentials (ERPs) [21,22]. Moreover, it provides a more ecologically valid assessment of brain activity and cognitive functioning by simulating real-life scenarios and enabling the investigation of functional connectivity patterns during specific cognitive processes [12]. The present study aims to perform EEG-based analysis in order to compare the functional connectivity in people diagnosed with SZ and healthy controls while they are focused on the content of a series of video clips with respect to when their attention goes away from the task at hand (mind-wandering) [23,24].

Conversely, much SZ research underscores the hypothesis that the disorder is underpinned by connectivity issues in various brain regions, emphasizing the role of disrupted functional connectivity in SZ’s pathophysiology [25,26,27,28]. While traditional studies have provided significant insights using average time series values to characterize functional connectivity [26,29], recent studies acknowledge the brain’s dynamic nature, advocating for a shift towards examining functional connectivity patterns across different time points [30,31]. Aligning with this, our work seeks to identify potential anomalies in SZ brain connectivity, utilizing both static (average of time series) and dynamic (temporal dynamics of functional connectivity) indicators.

The measures of graph theory, the mathematical study of networks, allow for the quantitative characterization of global and local topological properties within and between large-scale brain networks. The ability to analyze these networks with graph theory measures offers a psychologically meaningful research program capable of identifying critical changes in certain properties of the networks, both in normal cognitive function and in disordered states. Previous research on graph theory in schizophrenia has shown the sensitivity of these measures to capturing a disturbed balance between segregation and integration within functional brain networks [14,32,33,34,35]. In this paper, this approach will be adopted.

Considering all of the above from EEG data, the present study aims to use measures based on graph theory to obtain dynamic and static indicators of the functional connectivity of individuals diagnosed with SZ and healthy controls, both in situations in which they are focused on a task and when they are mind wandering (stimulus-independent and task-unrelated thoughts).

## 2. Materials and Methods

Our dataset was part of a large investigation designed to compare internally guided cognition states in individuals diagnosed with SZ and healthy controls [36]. The results to be presented in this paper have not been published, and the research questions are original. Since the sample description and the cognitive functioning assessment procedures used have been previously described in detail [36], in this section, those procedures are briefly described.

### 2.1. Participants

Our dataset was part of a large investigation designed to compare internally guided cognition states in individuals diagnosed with SZ and healthy controls [36]. The results presented in this paper (see Table 1) have not been previously published, and the research questions are original. Since the sample description and the cognitive functioning assessment procedures used have been previously described in detail [36], in this section, these procedures are only briefly described.

Cognitive functioning was measured using a Spanish adaptation of Screening for cognitive impairment in psychiatry [37]. SCIP-S allows for the detection of cognitive deficits in people with mental disorders, although it can also be used to assess the cognitive status of adults without a mental disorder. SCIP-S provides a score for each of the following subscales: immediate and delayed verbal learning, verbal fluency, working memory, and processing speed.

To assess psychopathology, a Spanish version [38] of the positive and negative syndrome scale [39] was used. PANSS is composed of 30 items that evaluate schizophrenic syndrome, each item being scored according to a scale of 7 points of severity (1: absence of the symptom, 7: presence with extreme severity).

### 2.2. Procedure

Data collection was conducted in two sessions in a laboratory at the Hospital Universitario San Agustín. During the first session, after signing the informed consent form, the participants were seated approximately 70 cm away from a computer screen. Next, the experimenter placed a set of 32 active electrode caps on the 10–20 system, keeping their impedance under 5 kOhm. The frequency of signal recordings was 500 Hz.

**Figure 1:**
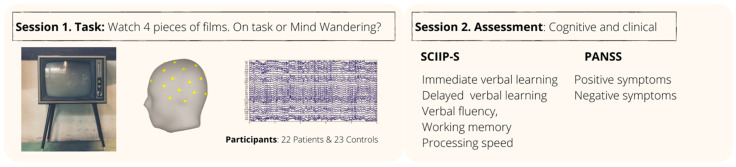
Data recollection procedure.

Participants were informed that they would watch four film clips and would be asked intermittently whether they were focusing on the content of the video or on their own unrelated thoughts. The movie would pause, approximately every 50 s, and then a message would appear on screen, where participants were asked to indicate whether they had been concentrating on the video (referred to as the “attention-to-task condition”) or whether they had stopped paying attention to the video and had been focusing on internal stimuli, such as thoughts, mental images, or memories unrelated to the task at hand (referred to as the “attention-to-mind condition”). A researcher, who could not be seen by the participant, recorded the verbal responses by entering a numerical code into the EEG recording. This message remained for about 10 s, which was the time the participants had to respond. After this time, the movie would resume until the next question. In a second subsequent session, participants underwent a cognitive assessment using the Spanish adaptation of the screen for cognitive disability in psychiatry (SCIP-S). Following this assessment, for participants who were patients, a clinician administered the Spanish version of the positive and negative syndrome scale (PANSS). A summary of the experimental setup is presented in Figure 1.

### 2.3. EEG Data Processing

For each participant, 20 clean EEG segments were selected, each with a length of 50 s, labeled according to the introspective answer of participants (mind wandering and on task). Data processing was performed using Brain Vision Analyzer software, EEGLAB [40], and MATLAB custom scripts. A bandpass filter was applied with cutoff frequencies of 0.5 and 30 Hz. Blinks, muscles, pop-up channels, and other artifacts were extracted using infomax runica independent component analysis (ICA) [41]. ICA components with artifacts were eliminated by visual inspection of the scalp topography, power spectra, and raw activity from all components.

### 2.4. Source Reconstruction and Parcellation

**Figure 2:**
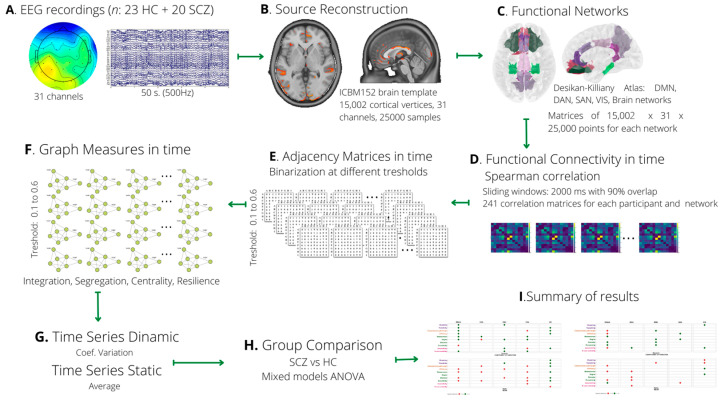
Workflow pipeline.

Each EEG trial contained 50 s of cortical activity distributed among the 31 channels we used for recording (in total, there were 32 channels, but as we have previously indicated, one of them was used as a reference). Since our sampling rate was 500 Hz, for each trial, we had 25,000 points for each of the 31 channels. A source model consisting of 15,002 current dipoles was used to calculate Kernel inversion matrices for each participant using sLORETA, implemented in Brainstorm [42]. We used the ICBM152 brain template, distributed with the Brainstorm package. The orientation of the dipoles was constrained to the cortex. The forward EEG model was computed for each subject using the boundary element method (BEM) implemented in the OpenMEEG model [43,44]. This source modeling process generated the complete matrices 15,002 × 25,000 × 31, where 15,002 are the virtual sources, 25,000 are the samples generated in the test (50 s at 500 Hz), and 31 are the channels. Figure 2 shows a diagram of the main steps in obtaining metrics, analysis, and results.

In order to parcellate the source model, the Desikan–Killiany atlas [45] available in Brainstorm was used. According to this parcellation scheme, we were able to define the following networks: default mode network (DMN), dorsal attention network (DAN), salience network (SAN), and visual network (VIS). Table S1 (see Table S1 in Supplementary Material) presents an exhaustive description of the regions of interest (ROI) that define each of these networks. In addition to the previously mentioned networks, a network (brain) was generated from all the ROIs, regardless of their network of origin. 

### 2.5. Functional Connectivity

For each of the networks (DMN, DAN, SAN, VIS, and Brain), the Spearman correlation coefficient between the electrical activity on its nodes was obtained. One of the main advantages of using EEG recordings is the high temporal resolution, which allows us to explore in detail the dynamics of temporal activation. For this purpose, a sliding window of 2000 ms with 90% overlap at each step was used, which produced a total of 241 functional connectivity matrices for each participant. The duration of the time series to which the sliding window was applied was 50 s, which corresponded to the time participants had been watching the videos.

The topological characteristics of the networks generated by binarizing the interregional correlation matrix depend on the threshold value chosen. This means that if a high threshold is used in the binarization process (i.e., the correlation is required to be high to be considered a functional link), this results in a low number of edges, i.e., the network has weak connectivity, which can lead to the disconnection of certain nodes. On the contrary, if a low threshold is used (i.e., low correlations will be accepted as indicators of functional relationships between nodes), then a network with denser connections but with a random topology is generated [32]. To address this relationship between network topology and the threshold used in binarization, [46] suggests that networks should ideally be evaluated across a wide range of thresholds. In this regard and following these recommendations, our study employed thresholds ranging from 0.1 to 0.6 at intervals of 0.1. Consequently, a total of 241 binary matrices were generated (which were unweighted and undirected) for each of the six thresholds explored. In this study, negative correlations were excluded from the network, consistent with previous research [46,47].

### 2.6. Graph Measures

The Brain Connectivity Toolbox was used to extract graph measures [46]. The metrics used in this study are explained in the Supplementary Material (see graph metrics section). Detailed explanations of each metric can be found in [46,48]. In this work, 10 graph theory measures were explored. These measures describe the basic properties of segregation (clustering coefficient, transitivity), integration (efficiency, characteristic path length), centrality (degree, betweenness, eccentricity, and diameter), and resilience (assortativity, k-core centrality) of brain networks [46,48].

### 2.7. Data Analysis

From the binary matrices, we calculated the dependent variables (graph measurements referred to in the previous section), both static (mean) and dynamic (coefficient of variation). The coefficient of variation (CV = (σ/μ) × 100, where: CV = coefficient of variation, σ = standard deviation of the dataset, and μ = mean of the dataset) is a statistical measure used to assess the relative variability or dispersion of data in comparison to its mean. It is particularly useful when comparing the variability of datasets with different units or scales, as it is a dimensionless ratio. In addition, the CV allows direct comparison of the relative variability between data sets or groups independently of their means. The data were analyzed using mixed-effects analysis of variance with group, threshold, and their interaction as fixed factors and participants as random factors. Analyses were performed in R (v 4.2.3) using the lmer() function of the lme4 package [49]. Post hoc comparisons and interaction analyses were made using the emmeans package [50]. We used the fdr procedure in order to control the error type I due to the number of comparisons [51].

## 3. Results

Due to the large number of dependent variables, to simplify the interpretation of the results and since our main interest was to compare functional connectivity in individuals diagnosed with schizophrenia with respect to the control group, the analyses were segregated into two categories, depending on the content of consciousness of the participants (mind wandering vs. on-task). The results for each condition are presented below.

Graph Measures during Mind Wandering and on-Task

**Figure 3:**
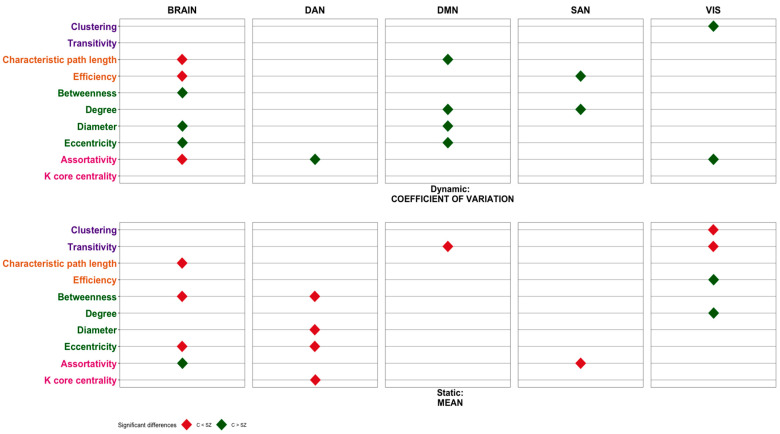
Functional connectivity comparisons between individuals diagnosed with schizophrenia (SZ) and controls (C) during mind wandering (MW) states in different networks. CV: coefficient of variation, M: mean, DAN: dorsal attention network, DMN: default mode network, SAN: salience network, VIS: visual network.

Figure 3 presents the significant differences found in each of the graph metrics to characterize functional connectivity between individuals diagnosed with schizophrenia and the group of healthy controls for each of the networks (DAN, DMN, SAN, VIS, and Brain). The top row of Figure 3 shows the differences found in the functional connectivity metrics using the dynamic information of time series (coefficient of variation) and in the bottom row using the static information (mean). In order to facilitate the understanding of the results in Figure 3, it has been represented with diamonds if differences between the groups appeared, regardless of whether the differences were found in one or several thresholds. Nevertheless, all the information regarding thresholds is presented in Table S2 (Supplementary Material). To facilitate the integration and interpretation of our results, the graph metrics have been grouped according to whether they indicate segregation, integration, centrality, or resilience. In the following figures, a color code has been assigned to each of these categories: segregation metrics (clustering and transitivity) have been coded in purple, integration metrics (characteristic path length and efficiency) have been coded in orange, centrality metrics (betweenness, degree, diameter, and eccentricity) have been coded in green, and resilience metrics (assortativity and k-core centrality) have been coded in pink. The tables presented are an attempt to summarize all the information obtained. All the details of the analyses carried out and additional figures can be found in [52].

**Figure 4:**
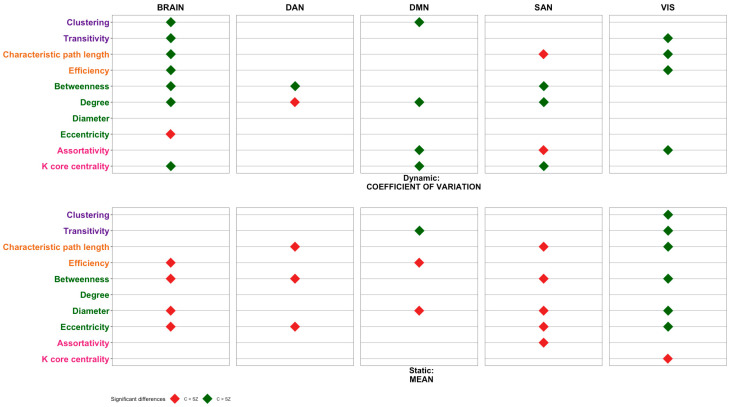
Functional connectivity comparisons between individuals diagnosed with schizophrenia (SZ) and controls (C) during on-task (OT) states in different networks. CV: coefficient of variation, M: mean, DAN: dorsal attention network, DMN: default mode network, SAN: salience network, VIS: visual network.

Figure 4 shows the significant differences between the groups (SZ vs. C) for each of the functional connectivity metrics in the different networks considered during on-task states.

Regarding static measures (mean, see lower row in Figure 3 and Figure 4), it can be seen that metrics indicating integration and centrality are lower in controls than in patients, in the global brain, and in the DAN network, especially during MW. These metrics are lower in controls, only in the on-task condition, and also in the SAN network (Figure 4, lower row). On the contrary, in the visual network, most metrics are higher in controls than in patients in the on-task condition (Figure 4, lower row). Differences between groups are more evident when dynamic measures (CV) are considered. It can be seen that centrality metrics are higher in controls than in patients, both in the brain network and in the DMN, when participants are mind-wandering (Figure 3, upper row). When they are on task, most metrics are increased in controls compared to patients, in the brain network, in the DMN, and in the visual network (Figure 4, upper row). Since the dynamic measure indicates the temporal functional organization of the brain networks, these results seem to indicate a more complex and rich function of brain networks in controls, which is especially evident when participants are on task.

## 4. Discussion

In this research, we have examined an ample number of metrics from brain networks in patients diagnosed with SZ and controls while they were in MW or were attending a task (OT). The results are complex, but two patterns clearly emerge. First, when the static measures (based on means) are considered, most of the functional connectivity metrics of the networks are increased in patients compared to controls (except in the visual network). However, when the dynamic measures are considered, patients with SZ showed reduced values in most metrics (segregation, integration, centrality, and resilience), reflecting a reduced number of dynamic states of brain networks. We will start discussing the results for the static measures and finish with the discussion of the dynamic measures.

### 4.1. Static Measures

Applying graph theory principles introduced by [46], the organization of the functional connectome can be described using two fundamental concepts of network structure: segregation and integration. Segregation pertains to how much a network displays clusters of regions for specialized processing, while integration refers to the effectiveness of information transmission across the entire network. Previous studies have identified irregularities in both the segregation and integration of the functional connectome in individuals diagnosed with schizophrenia, predominantly through the analysis of resting-state data [14,26,32,33,34,35]. Our results from the static data using naturalistic tasks, such as film viewing, agree with the previously mentioned results, particularly in the DMN, whose functioning has been found to be abnormal in schizophrenia [53]. Thus, in DMN during on-task states, participants with SZ showed significantly less transitivity (segregation) than controls. These results could indicate that in individuals with schizophrenia, these specialized modules that work together for specific tasks or functions might be disrupted, leading to a more interconnected and less distinct organization of these brain regions. This could result in difficulties with processing information efficiently and coordinating various cognitive functions. However, during MW episodes, the segregation pattern is reversed in the DMN, such that in the patient group, a significant increase in transitivity was observed. This increased transitivity suggests that the DMN is functioning in a more clustered manner during mind wandering in schizophrenia, reflecting a loss in the network’s ability to efficiently switch between different cognitive states, leading to a prolonged focus on internal cognition. These results are in line with previous results [36,54] showing that patients diagnosed with SZ showed more frequent and longer duration of MW episodes than controls.

Regarding integration measures, patients during OT showed higher path length (lower integration) than controls in DAN and SAN. These results are consistent with previous studies [26,28,33]. These altered patterns of functional connectivity may contribute to a weakness in salience processing that, in turn, contributes to the general deficits in external goal-directed behavior [55]. On the other hand, we found greater integration in patients than in controls in the DMN. Other studies have also found higher efficiency in individuals with an SZ diagnosis [26,56,57,58]. This could be related to increased self-focused rumination, heightened self-awareness, and possibly a reduced ability to disengage from internal thoughts. While some level of DMN connectivity is essential for self-related cognition, an overly integrated DMN might contribute to the excessive self-consciousness and cognitive rigidity often observed in schizophrenia. This deregulation between the integration-segregation dynamics in the systems in charge of salience processing and external goal-directed regulation (SAN and DAN, respectively), on the one hand, and the brain network engaged for internal thought (DMN), on the other hand, is consistent with the idea that network dysfunction is tightly linked to deficits in regulating salient information and maintaining the integrated self [59,60]. According to this hypothesis, the difficulties in distinguishing between self-representation and environmental salience processing may render the perception of neutral environmental stimuli as abnormally salient and could underlie some symptoms of schizophrenia [60].

Regarding centrality measures (betweenness, degree, diameter, and eccentricity), our results indicate that patients have significantly greater betweenness, diameter, and eccentricity than controls in the dorsal attention network (DAN), with this pattern being similar during both MW and OT episodes. The DAN is responsible for directing attention to relevant stimuli in the environment and for maintaining task-specific concentration. In addition, abnormalities in DAN connectivity, such as changes in betweenness and eccentricity, could result in disruption of communication between brain regions [61]. An elevated eccentricity signifies that certain nodes within the DAN are more distant or isolated from the network’s core. This could suggest that specific regions are not effectively integrating with the rest of the network. In SZ, an increased eccentricity might indicate difficulties in integrating and coordinating information between different parts of the brain, leading to disruptions in attentional processes and difficulties in integrating information from different brain areas [62].

The most apparent change between patients and controls, in terms of centrality parameters, occurs in the SAN during OT episodes versus MW episodes. Specifically, during mind wandering, SAN does not appear to differ between the SZ group and the control group in terms of centrality parameters. However, during OT episodes, patients showed more betweenness, diameter, and eccentricity than healthy controls. The salience network maintains and updates task demands in order to achieve the current behavioral goals. It has been associated with the automatic detection of significant events originating from both internal and external sources and might play a role in regulating the transition between the DMN (focused on internal cognition) and the DAN (focused on external stimuli) [63]. An increase in betweenness could reflect heightened efforts to direct attention to important stimuli. However, the increased diameter and eccentricity might hinder the network’s ability to fully process and prioritize these stimuli. This could lead to difficulties in accurately assessing the significance of events, emotions, or cognitive tasks. The observed alterations in betweenness, diameter, and eccentricity collectively suggest that the salience network’s ability to effectively integrate and process information is compromised. This could contribute to cognitive deficits such as impaired decision-making, difficulty regulating emotions, and challenges in perceiving and responding to environmental cues.

Regarding the centrality measures during OT episodes in the DMN, we found a significant increase in the diameter in the SZ group. These results could imply that the DMN is integrating information from a wider range of sources or regions and are consistent with the increased integration observed in the DMN and discussed previously. This enhanced centrality in the DMN promotes an abnormal integration of various cognitive and self-related aspects. Disturbances in self-referential processing in SZ associated with DMN hyperactivation have been reported in [64].

We also found a significant increase in assortativity in the SZ group during OT episodes in the SAN. Such an increase in network assortativity could be a sign of network dysfunction. If nodes with similar attributes are more connected, it might suggest that the network is becoming rigid and less capable of effectively processing diverse types of information. As mentioned previously, the SAN plays a crucial role in distinguishing between internally focused processes and external stimuli that require attention. If the SAN becomes rigid and fails to distinguish these processes effectively, this rigidity might lead to difficulties in properly modulating the network’s responses to different types of information [60].

### 4.2. Dynamic Measures

Until recently, most of the work in the field of the study of functional relations offered a stationary view of the relationships between network communities, as they tended to calculate functional relationships from measures based on signal averages. In recent times, neuroimaging research has started to unveil the dynamic organization of the brain’s functional organization using dynamic functional connectivity. This involves observing time-dependent correlations in the EEG signals between various brain regions over various time intervals. Furthermore, several studies have highlighted the importance of this phenomenon for cognitive processes and disease [65,66,67,68,69].

Neural dynamics lead to the formation of interconnected regions that appear and fade away over various time scales. A novel concept emerging from this idea is the notion of a functional repertoire of continuously revisited brain states [70]. The dynamics associated with this functional repertoire are believed to be fundamental for cognition [71] and are proposed to mirror the functional potential of a neural system [70,71].

Looking at the dynamic measures (coefficient of variation), our results indicate that individuals diagnosed with SZ show less variability in most measures of segregation, integration, centrality, and resilience, and this difference is especially marked in the OT condition. Reduced variability in these measures suggests that when individuals diagnosed with SZ are engaged in a task, their brain network shows less variability in these measures compared to individuals without SZ. Segregation refers to the degree to which specialized brain regions are functionally separated, while integration refers to the extent of communication and information flow between different brain regions. Centrality measures the importance of specific brain regions within the network, and resilience reflects the network’s ability to maintain its functionality despite perturbations. The reduced variability in these measures implies a narrower range of functional states in the brains of individuals diagnosed with SZ [72]. On the other hand, since the dynamic measure indicates the temporal functional organization of the brain networks, our results seem to indicate a more complex and richer function of brain networks in controls, especially evident when participants are on task.

### 4.3. Limitations

The first group of limitations has to do with access to clinical samples. In this regard, in our study, the recruitment process was not randomized, which introduces the possibility of selection bias. In addition, the sample size was relatively modest, which makes it necessary to be cautious when generalizing our results to patients from other centers with different ages, cultural levels, genders, treatments, and other variables that could be relevant in this context.

Furthermore, the graphical metrics depend on how the networks are delineated. In our investigation, the initial data came from EEG, which is characterized by restricted spatial resolution; source reconstruction was executed without customized anatomical data; and regions of interest (ROIs) were partitioned using an atlas. Therefore, caution is advised in drawing comparisons between the results of this study and those of investigations that established network definitions using alternative methodologies.

In addition, networks are subject to thresholds in order to eliminate spurious connections. When there are average between-group differences in functional connectivity, this thresholding procedure may bias the differences. To illustrate, let us consider the scenario in which patients show an overall decrease in connectivity. In such cases, if thresholds for the patient and control matrices are set to maintain the same density of connections, it is possible that more low-weight connections—potentially spurious in nature—would be included in the patient group data. This could result in a higher proportion of connections that are not really meaningful. Consequently, the resulting network could adopt a more random configuration in terms of topology (see detailed discussion in [72]. We would also like to point out that a reduction in structural connectivity need not lead to a reduction in functional connectivity. The brain adapts to pathology, so that dysfunction in one location may result in a compensatory increase in activity and/or connectivity in other areas [73]. Nevertheless, despite the complexity of our results and the aforementioned limitations, graph theory analysis is a fundamental tool in neuroscience research that facilitates the exploration of brain connectivity, organization, and function. It offers a very interesting perspective that helps to unravel the complex and intricate problems of brain networks and their role in cognition, behavior, and disease.

## 5. Conclusions

In this paper, we have presented the results of exploring by static and dynamic measures of graph theory functional connectivity in different networks using a naturalistic task such as the viewing of movie fragments compared with the states of distraction or mind wandering in a group of individuals diagnosed with SZ compared with a group of healthy controls.

Our findings revealed significant differences in the functional connectivity networks across various brain regions in the majority of the networks analyzed. These differences suggest that the brain connectivity patterns in individuals with schizophrenia diverge significantly from those in healthy controls, particularly during naturalistic tasks such as video viewing. These disparities in functional connectivity could shed light on the neural mechanisms underlying the cognitive and perceptual differences observed in individuals with schizophrenia.

Dynamic analysis, which considers the temporal variability of connectivity, further highlights the complexity of these differences. It underscores the importance of studying brain connectivity in a time-varying context to capture subtle fluctuations that may be overlooked in static analyses.

Our results contribute to a deeper understanding of the neural underpinnings of schizophrenia and suggest that disruptions in functional connectivity during naturalistic tasks may be a crucial aspect of the disorder. Future research should continue to explore the implications of these findings for diagnosis, treatment, and our broader understanding of schizophrenia’s neurobiological basis.

In summary, this study provides valuable insights into the differences in functional connectivity between individuals with schizophrenia and healthy controls, emphasizing the relevance of dynamic network analysis in uncovering the intricate patterns of brain connectivity associated with this psychiatric condition.

## Figures and Tables

**Table 1:** Demographical and clinical variables. Values expressed as mean ± standard deviation.

	SCZ	HC	Test, *p*-Value
N	22	23	
Age (y)	36.5 ± 10.2	38.8 ± 11.8	*U* = 222, *p* = 0.25 ^a^
Sex (M:F)	15:7	19:4	χ^2^ = 0.91, *p* = 0.33 ^b^
Education (Prim.:Second.:High)	5:13:7	2:14:7	χ^2^ = 2.12, *p* = 0.34 ^b^
SCIP-S—VLi	17.5 ± 4.2	20.8 ± 4.5	*U* = 150, *p* < 0.01 ^a^
SCIP-S—VLd	5.6 ± 2.4	6.6 ± 2.2	*U* = 213, *p* = 0.17 ^a^
SCIP-S—VF	13.4 ± 4.2	17.2 ± 4.2	*U* = 142, *p* < 0.01 ^a^
SCIP-S—WM	17.4 ± 4.2	19.2 ± 3.1	*U* = 207, *p* = 0.14 ^a^
SCIP-S—PS	8.1 ± 3.3	11.1 ± 3.2	*U* = 144, *p* < 0.01 ^a^
PANSS-P	14 ± 6.0		
PANSS-N	18.8 ± 7.6		
PANSS-G	32.4 ± 9.3		

SCZ: Individuals diagnosed with schizophrenia, HC: healthy control, VLi: iImmediate verbal learning, delayed verbal learning: VLd, verbal fluency: VF, working memory: WM, processing speed: PS, PANSS-P: positive symptoms, PANSS-N: negative symptoms, PANSS-G: general subscale. ^a^ Mann-Whitney test; ^b^ χ^2^ test.

## Data Availability

EEG data used in this research are available upon reasoned request. To protect the anonymity of the participants, clinical data will not be shared.

## Supplementary Materials

The following supporting information can be downloaded at: https://www.mdpi.com/article/10.3390/s23218722/s1, Graph metrics description; Table S1: Anatomic regions-of-interest (ROIs) included in the analysis, as derived from the Desikan Killiany atlas, and their affiliation to brain networks. Table S2. Significant differences between groups depend on the threshold.
