# Supplementary material for: Examining Neural Connectivity in Schizophrenia Using Task-Based EEG: A Graph Theory Approach
Source: Sensors (Basel). 2023 Oct 25;23(21):8722. doi: 10.3390/s23218722 (PMC10647645; doi:10.3390/s23218722)
Supplement: Supplementary file 1 [file sensors-23-08722-s001.zip › sensors-2645473-supplementary.pdf]

Supplementary materials

Graph metrics

The measures of graph theory, the mathematical study of networks, allow for the quantitative characterization of global and local topological properties within and between large-scale brain networks. The ability to analyze these networks with graph theory measures offers a psychologically meaningful research program capable of identifying critical changes in certain properties of the networks, both in normal cognitive function and in disordered states.

1. **Segregation**

Segregation indicates the level of which a network is partitioned into different regions to specialize neural processing. Clustering coefficients and transitivity are two related metrics that quantify the features of topological segregation in brain network.

Clustering. The clustering coefficient measures the degree to which nodes tend to form clusters or local neighborhoods. To calculate the clustering coefficient in an undirected graph, you can use the local clustering coefficient method.

Clustering Coefficient for a node = (Number of edges between neighbors of the node) / (k * (k - 1) / 2)

In this formula:

Number of edges between neighbors of the node refers to the count of edges that exist between the neighbors of a specific node.
k represents the degree of the node, which is the number of neighbors the node has.
k * (k - 1) / 2 denotes the total number of possible edges between the neighbors of the node.

Transitivity. Transitivity is the relationship between triangles and triplets in the network and is an alternative to the clustering coefficient. To determine the transitivity of an undirected graph, we can use the concept of triangle (set of three vertices connected by edges, forming a cycle of length three) and triplets (sets of three vertices). Let's denote the number of triangles as T and the number of triplets as Tri. The formula for transitivity in an undirected graph can be expressed as:

$$T = (3 * T) / Tri$$

This formula calculates the ratio of the number of triangles to the number of triplets in the graph, multiplied by 3. The multiplication by 3 is necessary because each triangle contributes to three different triplets. To compute the number of triangles (T) and triplets (Tri) in the graph, is necessary iterate over all possible sets of three vertices (triplets) and count the number of triangles formed by those vertices.

2. **Integration**

Integration refers to the efficiency of global communication in the network, which is usually measured by the characteristic path length of a network global efficiency that is inversely proportional to the characteristic path length of the network.

Efficiency. Is a measure that quantifies how efficiently information can flow between pairs of nodes in the graph. It is often used to assess the overall communication or connectivity efficiency of a network. The efficiency of an undirected graph can be calculated using the following formula:

Efficiency = (1 / Average shortest path length)

Characteristic path length. is a measure used in graph theory to quantify the average number of steps it takes to travel between pairs of nodes in an undirected graph. It provides insights into the overall efficiency of information transfer or communication within the graph. The characteristic path length is calculated using the following formula:

$$\text{Characteristic Path Length} = (1/(n*(n-1))) * \Sigma(d_{i,j})$$

Where:

n is the total number of nodes in the graph.
$d_{i,j}$ represents the shortest path length between nodes i and j.
$\Sigma(d_{i,j})$ sums up the shortest path lengths between all pairs of nodes.

A smaller characteristic path length indicates that information can be transmitted more efficiently within the network, as there are shorter paths connecting the nodes. On the other hand, a larger characteristic path length implies a less efficient network, with longer paths required for information to reach its destination.

3. **Centrality**

Centrality measures allow the identification of nodes that play an important role within the network. In this work we will use two measures, one based on the degree (degree centrality) and the other related to the average distance of a node from all the other nodes (betweenness centrality). On the other hand, it has been proposed eccentricity as a measure of the degree of compactness or homogeneity of a network. In addition, in this work we will calculate the diameter of the network to quantify the maximum eccentricity.

Degree. The degree of a node is a measure that quantifies the number of edges connected to that node. It represents the immediate connectivity of a node within the graph.

Betweenness. Quantifies the importance of a node based on its ability to act as a bridge or intermediary between other nodes. It calculates the number of shortest paths that pass through a particular node. The betweenness centrality of a node in an undirected graph can be calculated using the following formula:

Betweenness Centrality = Sum of (number of shortest paths passing through the node / total number of shortest paths)

Nodes with high betweenness centrality act as crucial bridges or intermediaries between other nodes in the graph. They play a significant role in connecting different parts of the network, facilitating communication and the flow of information.

Eccentricity. Measures the maximum distance or shortest path length between that node and any other node in the graph. It quantifies how far a node is from the furthest node in the graph. The

eccentricity of a node can be calculated by finding the longest shortest path from that node to any other node in the graph. A graph with a small range of eccentricities indicates a more homogeneous or compact structure, while a graph with a large range suggests a more dispersed or decentralized network.

Diameter. The maximum eccentricity across all nodes in the graph corresponds to the diameter of the graph. The diameter represents the longest shortest path between any pair of nodes in the graph, providing an overall measure of the graph's size or reach. The smaller the diameter, the shorter the paths between vertices, which means faster and more efficient communication in the network.

4. **Resilience**

Resilience of a network evaluates network vulnerability to insult[1]and can be measured by assortativity coefficient and by k-core centrality.

Assortativity. Is a measure that quantifies the tendency of nodes to be connected to other nodes with similar or dissimilar attributes. It examines the correlation between the attributes (such as degree, or any other node-specific characteristic) of connected nodes in the graph. The assortativity coefficient for an undirected graph can be calculated using the following formula:

$$\text{Assortativity Coefficient} = (\sum(e_{ii}) - (\sum(a_i * b_i)))/(1-(\sum(a_i * b_i)))$$

Where:

e_{ii} is the fraction of edges connecting nodes of the same attribute value (e.g., degree-degree).
a_i and b_i are the fractions of nodes with attribute value i.

A positive assortativity coefficient indicates a tendency for nodes with similar attributes to be connected. It suggests the presence of homophily, where nodes preferentially connect with others that share the same attribute values. This can imply the formation of communities or the reinforcement of similarities within the network.

K-core centrality. Identifies the central regions or core of a graph based on the concept of k-cores. A k-core is a subgraph in which all nodes have a degree of at least k. The k-core centrality of a node quantifies its importance within the k-core structure of the graph.

The k-core centrality of a node in an undirected graph can be calculated using the following steps:

Determine the maximum value of k (max k) such that a k-core exists in the graph.
For each value of k from 1 to max k, remove all nodes with degrees less than k from the graph.
Calculate the fraction of nodes that remain in the graph after removing the k-core.
The k-core centrality of a node is the maximum k value for which the node remains in the graph.

Nodes with high k-core centrality play a crucial role in maintaining the structural integrity of the graph. Removal of these nodes can lead to the disintegration of the k-core structure and the fragmentation of the graph into smaller components.

Table S1.
Anatomic regions-of-interest (ROIs) included in the analysis, as derived from the Desikan Killiany atlas, and their affiliation to brain networks.

**ROI**	**Network**

isthmuscingulate L	DMN
isthmuscingulate R	DMN
medialorbitofrontal L	DMN
medialorbitofrontal R	DMN
posteriorcingulate L	DMN
posteriorcingulate R	DMN
precuneus L	DMN
precuneus R	DMN
rostralanteriorcingulate L	DMN
rostralanteriorcingulate R	DMN
lateralorbitofrontal L	DMN
lateralorbitofrontal R	DMN
parahippocampal L	DMN
parahippocampal R	DMN
caudalanteriorcingulate L	DAN
caudalanteriorcingulate R	DMN
inferiortemporal L	DAN
inferiortemporal R	DAN
middletemporal L	DAN
middletemporal R	DAN
parsopercularis L	DAN
parsopercularis R	DAN
parsorbitalis L	DAN
parsorbitalis R	DAN
parstriangularis L	DAN
parstriangularis R	DAN
insula L	SAN
insula R	SAN
rostralmiddlefrontal L	SAN
rostralmiddlefrontal R	SAN
supramarginal L	SAN
supramarginal R	SAN
caudalmiddlefrontal L	SAN
caudalmiddlefrontal R	SAN
superiortemporal L	AUD
superiortemporal R	AUD
cuneus L	VIS
cuneus R	VIS
lateraloccipital L	VIS
lateraloccipital R	VIS
fusiform R	VIS
fusiform L	VIS
lingual L	VIS
lingual R	VIS

Table S2. Significant differences between groups depending on threshold

**Mind Wandering, BRAIN**	CV						MEAN					
	T1	T2	T3	T4	T5	T6	T1	T2	T3	T4	T5	T6
Clustering												
Transitivity												
Characteristic path length												
Efficiency												
Betweenness												
Degree												
Diameter												
Eccentricity												
Assortativity												
K core centrality												
c < sz; c > sz												

**Mind Wandering, DAN**	CV						MEAN					
	T1	T2	T3	T4	T5	T6	T1	T2	T3	T4	T5	T6
Clustering												
Transitivity												
Characteristic path length												
Efficiency												
Betweenness												
Degree												
Diameter												
Eccentricity												
Assortativity												
K core centrality												
c < sz; c > sz												

**Mind Wandering, DMN**	CV						MEAN					
	T1	T2	T3	T4	T5	T6	T1	T2	T3	T4	T5	T6
Clustering												
Transitivity												
Characteristic path length												
Efficiency												
Betweenness												
Degree												
Diameter												
Eccentricity												
Assortativity												
K core centrality												
c < sz; c > sz												

**Mind Wandering, SAN**	CV						MEAN					
	T1	T2	T3	T4	T5	T6	T1	T2	T3	T4	T5	T6
Clustering												
Transitivity												
Characteristic path length												
Efficiency												

Betweenness												
Degree												
Diameter												
Eccentricity												
Assortativity												
K core centrality												
c < sz; c > sz												
**Mind Wandering, VIS**	CV						MEAN					
	T1	T2	T3	T4	T5	T6	T1	T2	T3	T4	T5	T6
Clustering												
Transitivity												
Characteristic path length												
Efficiency												
Betweenness												
Degree												
Diameter												
Eccentricity												
Assortativity												
K core centrality												
c < sz; c > sz												
**On task, BRAIN**	CV						MEAN					
	T1	T2	T3	T4	T5	T6	T1	T2	T3	T4	T5	T6
Clustering												
Transitivity												
Characteristic path length												
Efficiency												
Betweenness												
Degree												
Diameter												
Eccentricity												
Assortativity												
K core centrality												
c < sz; c > sz												
**On task, DAN**	CV						MEAN					
	T1	T2	T3	T4	T5	T6	T1	T2	T3	T4	T5	T6
Clustering												
Transitivity												
Characteristic path length												
Efficiency												
Betweenness												
Degree												
Diameter												
Eccentricity												
Assortativity												
K core centrality												

c < sz; c > sz												
**On task, DMN**	CV						MEAN					
	T1	T2	T3	T4	T5	T6	T1	T2	T3	T4	T5	T6
Clustering												
Transitivity												
Characteristic path length												
Efficiency												
Betweenness												
Degree												
Diameter												
Eccentricity												
Assortativity												
K core centrality												
c < sz; c > sz												
**On task, SAN**	CV						MEAN					
	T1	T2	T3	T4	T5	T6	T1	T2	T3	T4	T5	T6
Clustering												
Transitivity												
Characteristic path length												
Efficiency												
Betweenness												
Degree												
Diameter												
Eccentricity												
Assortativity												
K core centrality												
c < sz; c > sz												
**On task, VIS**	CV						MEAN					
	T1	T2	T3	T4	T5	T6	T1	T2	T3	T4	T5	T6
Clustering												
Transitivity												
Characteristic path length												
Efficiency												
Betweenness												
Degree												
Diameter												
Eccentricity												
Assortativity												
K core centrality												

c < sz; c > sz

The tables above are an attempt to summarize all the information we have obtained. All the details of the analyzes carried out and additional figures can be found in the following link: https://osf.io/dj7rv/?view_only=eb76189f47f949919aaed7d2dffc0e2f

Iglesias-Parro, S., Soriano, M. F., Ibáñez-Molina, A. J., Pérez-Matres, A. V., & Ruiz de Miras, J. (2023, October 7). Examining Neural Connectivity in Schizophrenia Using Task-Based EEG: A Graph Theory Approach. Detailed analyses and figures. https://doi.org/10.17605/OSF.IO/DJ7RV
